# Parametric study of transcranial alternating current stimulation for brain alpha power modulation

**DOI:** 10.1093/braincomms/fcab010

**Published:** 2021-02-03

**Authors:** Beatrice P De Koninck, Samuel Guay, Hélène Blais, Louis De Beaumont

**Affiliations:** 1 Research Center, Hôpital du Sacré-Coeur de Montréal, CIUSSS du Nord-de-l’Île-de-Montréal Research Center (CRHSCM), H4J 1C5, Montreal, Québec, Canada; 2 Department of Surgery, Université De Montréal, H3T1J4, Montreal, Québec, Canada

**Keywords:** alpha power modulation, tACS, EEG, brain oscillations, non-invasive brain stimulation

## Abstract

Transcranial alternating current stimulation, a non-invasive brain stimulation technique, has been used to increase alpha (8–12 Hz) power, the latter being associated with various brain functions and states. Heterogeneity among stimulation parameters across studies makes it difficult to implement reliable transcranial alternating current stimulation protocols, explaining the absence of consensus on optimal stimulation parameters to modulate the alpha rhythm. This project documents the differential impact of controlling for key transcranial alternating current stimulation parameters, namely the intensity, the frequency and the stimulation site (anterior versus posterior). Phase 1:20 healthy participants underwent 4 different stimulation conditions. In each experimental condition, stimulation via 2 electrodes was delivered for 20 min. Stimulation conditions were administered at PO7-PO8 or F3-F4 at individual’s alpha frequency, or at individual’s theta frequency or sham. Stimulation intensity was set according to each participant’s comfort following a standardized unpleasantness scale (≤ 40 out of 100) and could not exceed 6 mA. All conditions were counterbalanced. Phase 2: participants who tolerated higher intensity of stimulation (4–6 mA) underwent alpha-frequency stimulation applied over PO7–PO8 at 1 mA to investigate within-subject modulation of stimulation response according to stimulation intensity. Whether set over posterior or anterior cortical sites, alpha-frequency stimulation showed greater increase in alpha power relative to stimulation at theta frequency and sham stimulation. Posterior alpha-frequency stimulation showed a greater increase in alpha power relative to the adjacent frequency bands over frontal and occipito-parietal brain areas. Low intensity (1 mA) posterior alpha stimulation showed a similar increase in alpha power than at high (4–6 mA) intensity when measured immediately after stimulation. However, when tested at 60 min or 120 min, low intensity stimulation was associated with significantly superior alpha power increase relative to high intensity stimulation. This study shows that posterior individual’s alpha frequency stimulation at higher intensities is well tolerated but fails to increase stimulation aftereffects recorded within 2 h of stimulation on brain oscillations of the corresponding frequency band. In sharp contrast, stimulating at 1 mA (regardless of phosphene generation or sensory perception) effectively and selectively modulates alpha power within that 2-h time window, thus validating that it as a reliable stimulus intensity for future studies. This study also shows that posterior alpha-frequency stimulation preferentially modulates endogenous brain oscillations of the corresponding frequency band. Moreover, our data suggest that posterior alpha-frequency transcranial alternating current stimulation is a reliable and precise non-invasive brain stimulation technique for persistent modulation of both frontal and occipito-parietal alpha power.

## Teaser

This study documents how key tACS parameters, namely the intensity, the frequency and the stimulation site, modulate alpha power via a within-subject paradigm involving four stimulation conditions. tACS after effects on alpha power were measured at fixed time points up to 120 minutes post-stimulation.

## Graphical Abstract

**Graphical Abstract:**
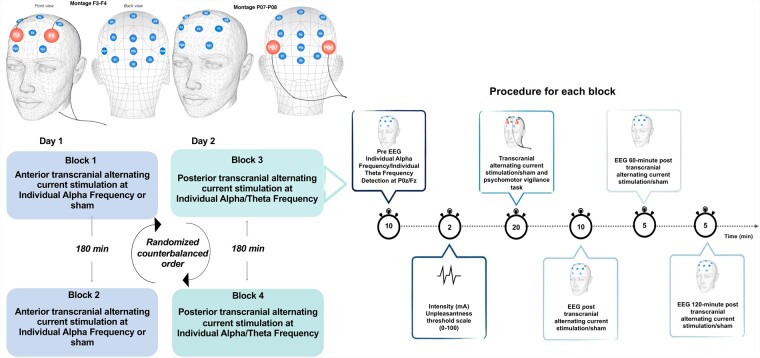

## Introduction

The growing interest for non-invasive neuromodulation research lies in part in its ability to impact brain function while concomitantly allowing direct measurements of related physiological changes. Brain oscillations are considered key to analyse and conceptualize cognitive processes (Başar, 2012; Gross, 2014; Başar *et al.*, 2016; Sadaghiani and Kleinschmidt, 2016). Clinical applications of non-invasive brain stimulation (NIBS) tools capable of modulating brain oscillations have also shown promising results (Başar *et al.*, 2013, 2016). Although it is generally agreed that the use of NIBS requires the implementation of double-blind and active/sham conditions (Brignani *et al.*, 2013), the lack of methodological gold standards is in part responsible for the concerning reproducibility failures currently undermining the translational value of these technologies.

Among different NIBS technologies, transcranial alternating current stimulation (tACS) stands out for its specificity of effect on brain oscillations. The possibility to enhance endogenous brain oscillations by the administration of an external sinusoidal current fixed at an analogous frequency through the scalp is identified as the main advantage of tACS over other NIBS (Paulus, 2011; Thut *et al.*, 2011; Antal and Paulus, 2013; Tavakoli and Yun, 2017). This enhancement of endogenous rhythm can be achieved at current strengths of very low intensity, hardly noticeable, as it is usually kept below photic or dermic sensory thresholds (Tavakoli and Yun, 2017), therefore preventing discomfort and allowing subject blindness to stimulation conditions. The efficacy of adjusted frequency and intensity parameters of tACS stimulation to modulate desired brain oscillations follows the assumption that the endogenous oscillatory activity interacts with oscillatory inputs originating from close to direct and/or intermediary sources (Buzsáki and Draguhn, 2004; Herrmann *et al.*, 2016; Romei *et al.*, 2016; Vosskuhl *et al.*, 2016; Tavakoli and Yun, 2017). A theoretical alternative to the presumed entrainment effects of tACS stimulation is that the latter technique generates neuroplastic changes that can be observed on frequency bands measured by electroencephalography (EEG), which can account for tACS’ aftereffects on EEG (Vossen *et al.*, 2015).

Although the underlying mechanisms of tACS remain the subject of ongoing debate and the characterization of optimal stimulation parameters are needed, the adjustment of tACS frequency to one’s endogenous peak alpha frequency has been used to modulate targeted EEG activity (Zaehle *et al.*, 2010, Neuling *et al.*, 2013b; Vossen *et al.*, 2015; Kasten *et al.*, 2016). This methodological approach is typically used to account for the known within and inter-individual variability in alpha activity (8–12 Hz) (Başar *et al.*, 2001; Başar, 2012; Mierau *et al.*, 2017; Negahbani *et al.*, 2018). To date, modulation of endogenous alpha activity was the target of most tACS studies in part due to its known implications in various sensory functions, cognitive processes and vegetative functions (Başar, 2012; Thut *et al.*, 2012; Thut, 2014). Modulation of alpha activity has shown great potential in improving cognitive function as well as in providing therapeutic relief for alpha-related brain conditions and pathologies (Montez *et al.*, 2009; Zaehle *et al.*, 2010; Başar, 2012; Başar *et al.*, 2013; Thut, 2014).

In addition to adjusting for tACS stimulation frequency, intensity of stimulation is another key experimental manipulation that currently fails to be addressed scientifically. One of the main caveats in the determination of optimal tACS stimulation intensity is that typically used stimulation parameters are based on safety considerations (Antal *et al.*, 2017), with little concern for their actual efficacy in humans. This is surprising considering that carefully increasing tACS stimulation intensity was identified as a potentially crucial element in optimizing tACS protocols (Antal *et al.*, 2017; Voroslakos *et al.*, 2018). To date, most studies have been concerned with stimulating at phosphene subthreshold intensities despite phosphene generation being completely harmless for human brain health (Zaehle *et al.*, 2010, Neuling *et al.*, 2013b; Vossen *et al.*, 2015; Kasten *et al.*, 2016; Antal *et al.*, 2017; Gundlach *et al.*, 2017). Indeed, studies have shown that phosphene induction under tACS stimulation typically occurs at very low stimulation intensities (i.e. below 1 mA), particularly so when stimulating over frontal brain areas (Kanai *et al.*, 2008; Schutter and Hortensius, 2010). Under such low intensity stimulation, however, one could question the effectiveness of tACS stimulation protocols given the known demonstrated soft tissue and bone attenuation of current flow of up to 75% (Voroslakos *et al.*, 2018). In a recent attempt to investigate the effects of increasing tACS stimulation intensity, Voroslakos *et al*. (2018) used a particular circular electrode disposition aiming to generate intersectional short pulses. This innovative methodological approach allowed the administration of higher current intensity via the summation of multiple smaller electric fields and through short pulses of induced currents (Voroslakos *et al.*, 2018). In the latter study, significant increase in alpha band amplitude was found only for current density exceeding 4.5 mA for the tested sample of healthy individuals. No detectable change in spectral power was observed for current intensities below 2 mA (Voroslakos *et al.*, 2018). However, no assessment of side effects nor tolerability was disclosed for such intensities (Voroslakos *et al.*, 2018). Therefore, even though the latter methodology differs from continuous administration of the current, this study raises the possibility that increasing stimulation intensity can be achieved among healthy humans.

Other than the general rule of thumb suggesting that tACS should be positioned over brain regions involved in a targeted brain function or near generators of targeted endogenous brain oscillations, very little scientific validation of the site of tACS stimulation is provided (Kanai *et al.*, 2010; Zaehle *et al.*, 2010, Neuling *et al.*, 2013b; Helfrich *et al.*, 2014; Vossen *et al.*, 2015; Kasten *et al.*, 2016; Gundlach *et al.*, 2017). Moreover, only a few studies have included measurements of alpha modulation over control cortical sites outside brain generator range. This is surprising considering that the latter methodological approach is routinely used as a standard validity assessment in other NIBS such as repetitive transcranial magnetic stimulation studies (Uddin *et al.*, 2006; Keuken *et al.*, 2011; Scheeringa *et al.*, 2012; Saiote *et al.*, 2013; Forsyth *et al.*, 2014).

Given the provisional state of knowledge on tACS, the impact of tACS stimulation at alpha frequency on other frequency bands is understudied. In a recent study by Helfrich *et al*. (2014), investigators found a specific increase in alpha power compared to theta and beta-band power following a 20-min tACS session at 10 Hz administered over the occipito-parietal cortex (Helfrich *et al.*, 2014). Whether similar alpha-specific effects are obtained under different stimulation parameters conditions remains to be tested.

This study therefore seeks to provide a rigorous investigation toward defining optimal stimulation parameters for alpha wave modulation using tACS. In this methodological investigation of tACS stimulation, we sought to exert control over six key confounding variables of tACS to assess its validity in inducing alpha-specific effects.

### Frequency of stimulation

In order to control for stimulation frequency effects on changes of alpha activity, our study design contrasted the effects of alpha versus theta tACS stimulation administered over occipito-parietal cortices. Alpha activity is known to originate from occipito-parietal cortex (Schürmann and Başar, 2001, Romei *et al.*, 2008a; Başar, 2012; Helfrich *et al.*, 2014) and the latter brain region corresponds to the cortical site from which most studies have shown alpha entrainment effects (Zaehle *et al.*, 2010; Helfrich *et al.*, 2014; Vossen *et al.*, 2015; Kasten *et al.*, 2016; Gundlach *et al.*, 2017). Although to a lesser extent than alpha activity, theta frequency is also recorded from occipito-parietal cortex, but theta generators are rather located over temporal cortical areas (Buzsáki, 2002; Munro Krull *et al.*, 2019). In light of the well-documented effects of alpha tACS on alpha activity, we hypothesized that tACS frequency set at individual’s alpha frequency (IAF) and delivered over generator site would be more effective in increasing alpha power relative to both tACS frequency set at Individual Theta Peak (ITF) delivered over alpha generator site and sham stimulation.

### Site of stimulation

The present experiment also examined the effects of stimulation sites on alpha activity generation, where alpha tACS is applied: (i) over occipito-parietal cortices, where alpha oscillations are thought to originate and where alpha activity is dominant and (ii) over anterior cortical areas, where alpha activity is recorded, but not dominant (Schürmann and Başar, 2001, Romei *et al.*, 2008a; Helfrich *et al.*, 2014). To further investigate potential effects of alpha tACS stimulation outside alpha generator range, we also proposed to contrast alpha activity recorded from the same EEG electrodes when tACS is delivered over anterior brain areas relative to a sham condition. We hypothesized that tACS frequency set at IAF would result in an increase in alpha power specific to the stimulation site. Additionally, alpha tACS delivered over the occipito-parietal cortical site would induce greatest increase in alpha power compared to anterior tACS.

### Intensity of stimulation

Potential differential effects of stimulation intensity on the modulation effect of alpha power by tACS are mostly the subject of theorizations and remain a challenge to address (Antal *et al.*, 2017; Voroslakos *et al.*, 2018). However, for the analogous method of transcranial direct current stimulation, a non-linear relationship to cortical excitability was reported as intensity increased (Batsikadze *et al.*, 2013). To address this dilemma with tACS, we conducted a second study phase where participants who underwent high intensity (4–6 mA) within comfort levels [visual analogue scale (VAS) of unpleasantness] were submitted to an equivalent second session of IAF tACS set at low intensity (< 1 mA). We hypothesized that high intensity IAF tACS would induce a greater increase in alpha power than low-intensity IAF tACS.

### tACS modulation specificity on frequency bands

Additionally, to test the specificity of alpha tACS modulation effects, recorded endogenous alpha activity from frontal and occipito-parietal montages was compared to that of adjacent frequency bands (i.e. theta and beta). We hypothesized that stimulating at IAF frequency would induce an overall increase in alpha power greater than that of adjacent theta and beta band power.

### Alpha power aftereffects

tACS aftereffects on alpha spectral power have been reported to last up to 70 min following continuous 20-min tACS protocol set at IAF (Kasten *et al.*, 2016). However, time lapses of alpha aftereffects have usually been documented under the 60-min period (Zaehle *et al.*, 2010, Neuling *et al.*, 2013b; Vossen *et al.*, 2015). This led us to hypothesize that under analogous continuous tACS protocol, spectral power modulation effects would not outlast the 60-min period following IAF-tACS.

### Comfort assessment

Finally, we tested whether alpha tACS effects and comfort would be maintained despite increasing stimulation intensity up to 6 mA based on an established 40/100 VAS score of unpleasantness rather than the typical phosphene self-reports (Boitor *et al.*, 2016), following a staircase procedure adjustment (Jensen *et al.*, 2003; Hawker *et al.*, 2011). One may argue that this approach creates bias in the sham condition, although our reasoning is that even at sub-threshold intensities set prior to stimulation, phosphenes are experienced in most cases when a continuous current is administered (Romei *et al.*, 2008a). In regard to these careful adjustment steps, we hypothesized that stimulation at higher intensities (> 2 mA) would be well tolerated when set according to comfort levels.

## Materials and methods

### Participants

Twenty healthy volunteers took part in this study. The study sample for phase one consisted of 20 participants [10 females, mean age of 25.40 years (SD = 3.73)]. Years of education varied from 11 to 23, with a mean of 17 years (SD = 3.11). The second phase of our study implicated 11 participants [6 females, mean age of 26.09 years (SD = 3.78)]. Years of education varied from 11 to 23, with a mean of 17 years (SD = 3.35). This study was approved by the Research Ethics Committee of the Hôpital du Sacré-Coeur de Montréal and all participants provided written informed consent before testing. Volunteers received financial compensation for their participation.

Participants were healthy and did not take any medication at the time of testing. Exclusion criteria included: (i) a history of neurological disorder (i.e. stroke, encephalopathy, seizure disorder, brain tumour and traumatic brain injury); (ii) a history of psychiatric illness; (iii) evidence of a developmental learning disability or attention deficit hyperactivity disorder; (iv) a history of alcohol and/or substance abuse and (v) clinically relevant indices of anxiety (Beck Anxiety Inventory ≤ 9) (45) or depression (Beck Depression Inventory II ≤ 13) (46) at the time of testing. All volunteers had no other contraindication to tACS.

### EEG

The experiment was performed in a dimly lit, sound-attenuated room [Research Center, Hôpital du Sacré-Coeur de Montréal, CIUSSS du Nord-de-l’Île-de-Montréal (CRHSCM), Montreal, Canada] for optimal EEG recordings. Constant EEG monitoring throughout the entire experimental protocol was performed from a room adjacent to the testing cabin via a camera and a microphone installed in the cabin.

Resting EEG was recorded from 15 electrodes positioned according to the 10–10 International System of electrode placement (Helfrich *et al.*, 2014). Grass Reusable 10 mm Gold Cup Surface Electrodes were mounted by a certified medical electrophysiologist. Two different recording electrode montages were set up according to the position of the tACS stimulator. For the first testing day, the tACS stimulator was positioned over F3 and F4 electrode sites (frontal tACS). In addition to electrodes common to both tACs montages—namely Fp1, Fp2, Fpz, C3, C4, Cz, P3, P4, Pz, POz, O1, O2 and Oz—EEG recording electrodes were positioned over PO7 and PO8 electrode sites. On the second testing day, tACS stimulation was applied through stimulating electrodes positioned over PO7 and PO8 electrode sites (occipito-parietal stimulation) while additional recording electrodes were placed over F3 and F4 electrode sites. Reference electrodes were placed on mastoids. Acquisition of EEG signal was made using a 32-channel Grass polygraph (Rhode, Island, USA) and (sensitivity, 7 μV/mm; bandpass, 0.3–100 Hz). EEG signal was digitized at a sampling rate of 256 Hz using the Harmonie software (Harmonie, Stellate Systems, Montreal, QC, Canada). Electrode impedance was kept below 5 kΩ.

EEG data preprocessing was performed off-line with Brainstorm (Tadel *et al.*, 2011). A high-pass filter was set at 0.1 Hz, without the application of a low-pass filter. Instead, we used a 60 Hz notch filter to filter recurring EEG data contamination. Semi-automatic artifact rejection was then performed using the *Brainstorm’*s detection of custom event feature on EEG data, set to detect 1–7 Hz artifacts of eye movements, blinks, movements or dental work (Tadel *et al.*, 2011, 2020). After automatic artifact detection was completed, we performed a manual verification to validate data preprocessing. Recordings from the eyes-open condition were finally segmented into 2-s epochs for subsequent data analysis.

### tACS

All participants were administered tACS on their scalp via Pro Carbon IFC electrodes (5.8 cm diameter) positioned over aforementioned electrode sites with a layer of conductive electrode gel Signagel (Parker labs, USA) and fixed by EC2 Adhesive and conductive cream (Natus, Austria) at the circumference. The tACS device (DS4 Bi-Phasic Current Stimulator, Digitimer, United Kingdom) was controlled through a Matlab (R2014b) software on a portable computer setup for the protocol.

**Figure 1:**
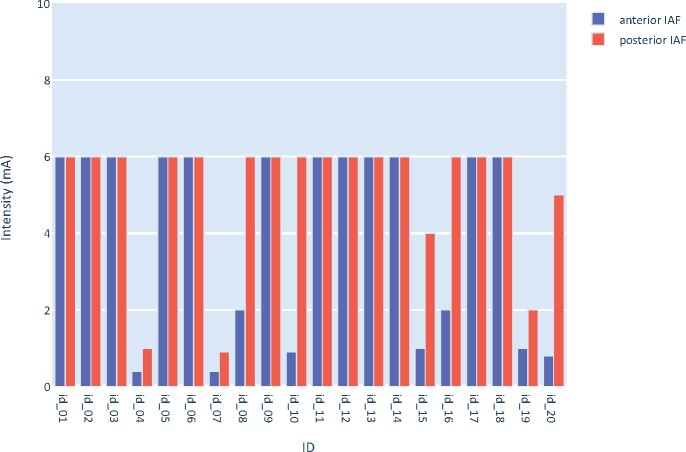
Intensities (mA) for each participant for IAF tACS conditions.

For each stimulation session, stimulation parameters including individual stimulation frequency and intensity were individually adjusted. For the occipito-parietal tACS montage, stimulation frequency was determined according to one’s IAF (8–12 Hz) measured from the POz electrode and extracted from the resting, eyes-open EEG condition using Fast Fourier Transforms. ITF as stimulation frequency (4–8 Hz) was also measured from the POz electrode and extracted from the resting, eyes-open EEG condition using Fast Fourier Transforms. For the anterior tACS montage, IAF was determined according to individual alpha peak (IAP) frequency (8–12 Hz) measured from the Fz electrode and extracted from the resting eyes-open EEG condition using Fast Fourier Transforms. For both occipito-parietal and frontal tACS montages, stimulation intensity was set according to the level of comfort of participants with a maximal intensity of 6 mA (see Fig. 1 for an overview of intensities according to IAF conditions), up to a predetermined cut off of 40/100 on the VAS unpleasantness (Kanai *et al.*, 2008; Schutter and Hortensius, 2010; Gundlach *et al.*, 2017). Participants were simply asked to describe sensations experienced during tACS stimulation, which were reported as tingling, pulsations/pulses, pins and needles and in some cases, warmth. Follow-ups on potential side effects were conducted following conditions and at the end of each day.

### Procedure

**Figure 2:**
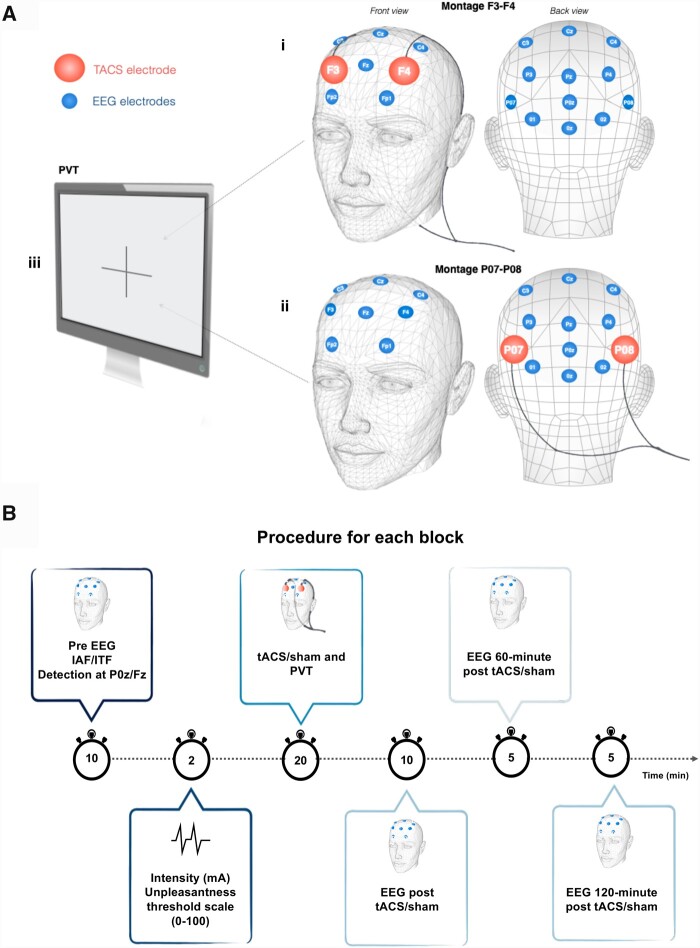
Overview of the protocol. Two different tACS montages were used for the protocol in A. EEG recordings took place before and after tACS. Day one included two sessions of tACS stimulation delivered over F3F4 (EEG International System, 10–20) with either adjusted frequency to IAP or sham stimulation in i. Day two included two sessions of tACS delivered over PO7PO8 (EEG International System, 10–10) with either adjusted frequency to Individual Alpha Peak (IAF) or ITF in ii. Participants underwent a psychomotor vigilance task on a computer during the 20-min tACS in iii. Four stimulation blocks on a 2-day testing period with a different montage for each day (anterior versus posterior) in **B**. The first day consisted of either sham or anterior IAF tACS, and the second day consisted of either posterior IAF or ITF tACS. A total of 180 min separated each stimulation/sham block. Each block included: 10-min at-rest EEG recordings conducted before and after 20-min tACS sessions to assess IAF or ITF induced effects on alpha activity. Intensity was set according to VAS (0–100) and remained within comfort levels. Following tACS, 5-min at-rest EEG recordings were collected at 60 min and 120 min post-stimulation

An overview of the protocol is provided in Fig. 2A. Participants were administered each of the four tACS stimulation sessions, each lasting for a maximum of 1 h. Two sessions were planned per day with a fixed, 3-h pause between sessions. This methodological decision rests on previous studies showing aftereffects lasting up to 70 min post-tACS for a 20-min protocol (Kasten *et al.*, 2016). Therefore, delaying sessions by a fixed 3-h interval controls for potential carry-over effects of tACS stimulation. Our single blind (blinding of participants) study design included three active tACS conditions and one sham condition. For the sham condition, identical methodology was applied, but stimulation current was only administered via a ramp up lasting 30 s, after which the software stopped the stimulation. Participants were given instructions preceding the experiment, with explanations on the various sensations and stimulation patterns they could experience. Intensity was first set at 0.1 mA and reached 6 mA for the majority of participants. The duration of experimental conditions was fixed at 20 min. The number of cycles administered varied according to individual stimulation frequency (α-cycles; alpha cycles or θ-cycles; theta cycles). Before initiating tACS stimulation, participants were instructed that stimulation parameters could vary across testing sessions and that this could affect their perception of the stimulation. For each condition of this protocol, sensations were documented according to the VAS of unpleasantness once the threshold intensity was determined, so prior to tACS/sham stimulation.

Before each tACS stimulation block, resting state EEG data were collected, during which volunteers were asked to keep their eyes open for 3 min, then closed for 30 s. These segments were repeated three times, for a total recording period of 10 min. Only the 3-min eyes-open segments were used in this study. Following each tACS stimulation block, two additional 5-min at-rest EEG acquisitions were performed both 60 min and 120 min following the completion of stimulation sessions (see Fig. 2B for a schematic representation of the study protocol). These additional at-rest EEG segments were acquired to compare effects of spectral power modulation over time. Note that the latter segments were shortened to reduce undesired effects of sleepiness and discomfort associated with prolonged study duration. These resting EEG data collections were acquired under similar recording conditions, with 2 min of eyes-open followed by 30 s of eyes-close, repeated twice. For these 5-min EEG recordings, only the eyes-open segments were used for analyses. Block randomization was used in order to counterbalance order of stimulation conditions within each day and across participants. Participants were all tested according to the same time frames. A psychomotor visual vigilance task (Dorrian *et al.*, 2004) was administered during the 20-min tACS blocks in order to control for the known state-dependent effects associated with alpha oscillations (Dinges and Powell, 1985; Dorrian *et al.*, 2004; Lim and Dinges, 2008; Mierau *et al.*, 2017) as well as for fatigue and vigilance (Craig *et al.*, 2012; Tanaka, 2015). The task was identical across stimulation conditions.

### EEG analysis

EEG analyses were performed with Brainstorm (Tadel *et al.*, 2011), an open-for-download software under the GNU general public licence (http://neuroimage.usc.edu/brainstorm, 21 January 2021, date last accessed) and Matlab (version 2018a, MathWorks, Natick/USA).

### Modulation of brain oscillations via tACS

#### Individual frequency peak determination

Prior to stimulation, the evaluation of the individual frequency peak was conducted on the pre-tACS recordings. Fast Fourier Transforms were performed on 2-s segments to obtain averaged spectras, similar to established procedures (Zaehle *et al*., 2010). The prominent frequency peak (either theta or alpha, depending on the stimulation condition) was then visually detected and later set as the tACS stimulation frequency. The same IAP determination procedure was followed for the sham stimulation condition.

#### EEG data analyses

Spectral power analyses were performed on three frequency bands of interest (theta, alpha and beta). Analysis procedure was kept constant across tACS conditions. The first 3-min, eyes-open, at-rest recordings following tACS stimulation were segmented into 2-s epochs in order to quantify EEG activity. Mean spectral power was obtained using Welch’s method (The MathWorks, 1994; Hayes, 1996; Stoica and et Moses, 2005) by frequency bandbands; theta (4–8 Hz), alpha (8–12 Hz) and beta (8–32 Hz). Results were then averaged across all epochs for each frequency band.

### Analyses of tACS aftereffects over time

Given previous demonstrations of tACS aftereffects lasting up to 70 min, we sought to measure potential tACS stimulation effects on alpha oscillations both 60-min and 120-min post-stimulation.

The possible modulation effects of alpha at 60 min and 120 min post-tACS stimulation were compared to those of theta and beta frequencies power change ratios. Identical signal pre-processing steps were performed with these resting 5-min EEG data samples, using only eyes-open segments for a total of 3 min of recording.

### Analyses of within-subject stimulation strength intensity effects

We also investigated whether stimulation intensity modulated alpha power during a second study phase (phase 2), by comparing a subsample (*N* = 11) who tolerated high intensity of IAF stimulation 4–6 mA over PO7PO8. This subsample underwent a supplementary block of PO7PO8 IAF tACS stimulation on a separate day, but set at 1 mA, therefore allowing tangible comparisons with higher intensity tACS effects on alpha power modulation.

### Statistical analyses

For all statistical analyses, parametric tests were used (R Core Team, 2019; The Jamovi Project, 2019) on recorded EEG signals pooled according to brain regions (i.e. frontal, central and occipito-parietal). The *anterior* electrode pool consisted of FP1, FP2 and Fz electrodes; the *central* electrode pool consisted of C3, C4 and Cz electrodes and the *occipito-parietal* pool consisted of POz, O1, O2 and Oz electrodes. Averaged within-subject EEG activity change ratios prior to and after tACS stimulation block [i.e. (Post-Pre)/Pre] were performed across frequency bands of interest. To this end, we used Python (Python Software Foundation, 2020) for data wrangling and preprocessing and jamovi (54) for statistical analyses. The GAMLj (Gallucci, 2019) package was used to perform linear mixed-effect models (LMMs) for which the assumption of normality of distribution of residuals was met for all analyses (Meteyard and Davies, 2019). Analysis of the standardized residuals extracted from the models were performed to verify normality of distribution. Presence of outliers was assessed for standardized values (z-scores) exceeding the absolute value of 3.29. Identified outliers were removed from the data set, for a maximum identified removal of 1.07%. In the presence of significant interactions, assessment with a least square means by lsmeans (Russell, 2019) was performed to fixed factors. Post-hoc tests or contrasts were all applied with a Bonferroni adjustment for multiple comparisons. Statistical significance threshold was set to 0.05.

### Data availability statement

The data that supports the findings of this study are available from the corresponding author, LDB, on request.

## Results

**Phase one.** An overview of descriptive statistics for frequency, intensity and unpleasantness scores for each montage and condition is provided in Table 1. Mean scores of unpleasantness (out of 100) were [anterior IAF tACS (*M* = 21.00, SD = 12.37); posterior IAF tACS (*M* = 19.23, SD = 10.46); posterior ITF tACS (*M* = 17.00, SD = 11.45) and sham (*M* = 18.33, SD = 9.76)]. Mean individual stimulation frequency were [anterior IAF tACS (*M* = 9.48 Hz, SD = 1.26); posterior IAF tACS (*M* = 9.63 Hz, SD = 1.09); posterior ITF tACS (*M* = 6.30 Hz, SD = 0.91) and sham (*M* = 9.48 Hz, SD = 1.35)]. Mean intensities were [anterior IAF tACS (*M* = 4.03 mA, SD = 2.51); posterior IAF tACS (*M* = 5.15 mA, SD = 1.74); posterior ITF tACS (*M* = 4.73 mA, SD = 1.90) and sham (*M* = 4.45 mA, SD = 2.32)]. Reported sensations by participants were tingling, pulsations, light scratching and warmth. A repeated measure LMM was also performed to compare the baseline spectral power of alpha band between conditions for each participant. We included spectral power of alpha as the dependent variable, stimulation conditions (posterior ITF, posterior and anterior IAF and sham) as the fixed effect and the subject ID as a random effect. No significant difference was found between conditions for each participant on their individual alpha power at baseline, *F*(3,3.82^−11^) = 0.09, *P *=* *1.00.

**Table 1:** Descriptive statistics for frequency (Hz), intensity (mA) and unpleasantness (VAS) according to tACS montage and condition

	Montage	Condition	Frequency (Hz)	Intensity (mA)	Unpleasantness (VAS)
Phase 1, anterior tACS					
Mean	F3F4	F	9.48	4.03	21.00
		S	9.48	4.45	18.33
Standard deviation		F	1.26	2.51	12.37
		S	1.35	2.32	9.76
Phase 1, posterior tACS					
Mean	PO7PO8	A	9.63	5.15	19.23
		T	6.30	4.73	17.00
Standard deviation		A	1.09	1.74	10.46
		T	0.91	1.90	11.45
Phase 2, posterior tACS					
Mean	PO7PO8	A	9.36	1.00	10.64
Standard deviation			1.27	0.00	9.88

A = posterior IAF tACS; F = anterior IAF tACS; S = Sham stimulation condition; T = posterior ITF tACS.

**Phase two.** An overview of descriptive statistics for frequency, intensity and VAS unpleasantness scores for each montage and condition is provided in Table 1. Mean scores of unpleasantness (VAS) was 10.64 out of 100 (SD = 9.88). Mean individual stimulation frequency was 9.36 (SD = 1.29) and mean intensity was 1.00 mA (SD= 0.00). Reported sensations by participants were tingling, pulsations, light scratching and warmth.

### Frequency of stimulation

#### Posterior α-tACS versus θ-tACS

**Figure 3:**
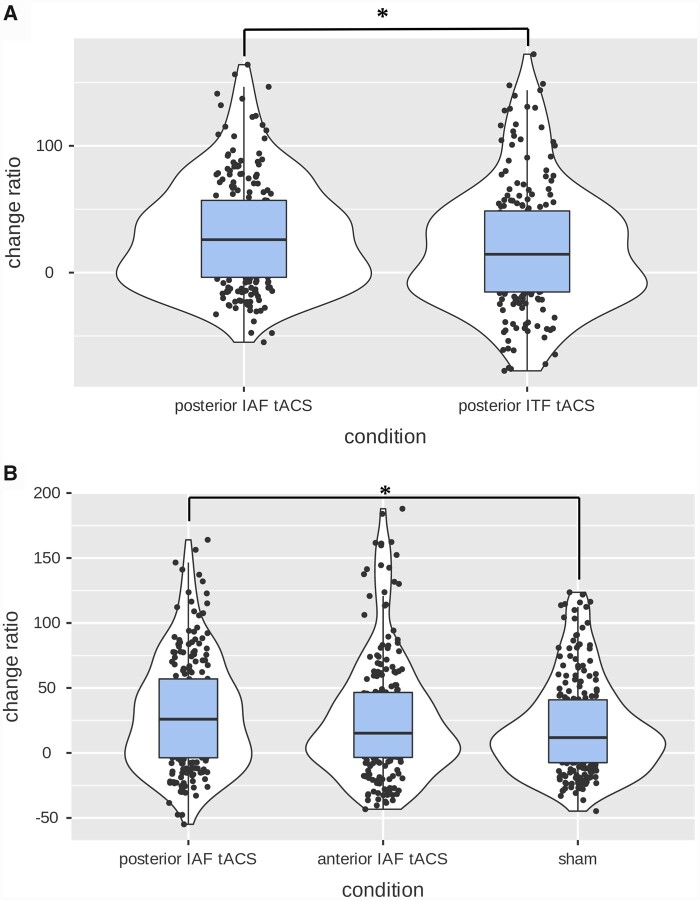
Significant greater change ratio of spectral alpha power for posterior IAF tACS than posterior ITF tACS and sham stimulation condition.

A first LMM was conducted to investigate stimulation effects within minutes following posterior tACS stimulation at IAF tACS stimulation versus ITF tACS stimulation on α-band power with EEG electrodes pooled over frontal, central and occipito-parietal regions. Refer to Table 2 illustrating this model. We included change ratio (%) as the dependent variable and added fixed effects of condition and electrode pool, as well as the interaction between condition and electrode pool. We included subject id as a random effect. The interaction between condition (IAF versus ITF) and electrode pool (frontal, central and occipito-parietal) on alpha power did not reach statistical significance, *F*(2, 404) = 1.40, *P *=* *0.25. No significant difference was found between electrode pools on alpha power, independently of condition, *F*(2,404) = 0.22, *P *=* *0.80. However, change ratios did reach statistical difference between main effect of tACS conditions, as IAF induced an overall significantly greater alpha power change ratio than ITF [IAF (*M* = 31.96%, SD = 45.24) and ITF (*M* = 19.86%, SD = 49.78)], *P *=* *0.03 (Fig. 3A). Essentially, for a fixed occipito-parietal tACS montage, the posterior IAF condition induced greater increase of alpha power than the ITF condition, without distinction between electrode pools.

**Table 2:** LMM analyses results for phase 1: fixed effects parameters estimates

Names	Effect	Estimate	SE	Lower 95% CI	Upper 95% CI	*df*	*t*	*P*
Phase 1: Frequency of stimulation, posterior IAF tACS versus ITF tACS								
(Intercept)	(Intercept)	24.98	5.09	15.00	34.97	19.51	4.91	< 0.001
electrode_pool1	Central-frontal	–2.99	5.39	–13.56	7.56	404.44	–0.56	0.58
electrode_pool2	Occipito-parietal-frontal	0.00	4.81	–9.42	9.42	404.48	4.21^-4^	1.00
condition1	T—A	–8.73	4.12	−16.80	–0.67	406.23	–2.12	0.03
electrode_pool1 * condition1	Central-frontal * T—A	–1.95	10.77	–23.06	19.17	404.44	–0.18	0.86
electrode_pool2 * condition1	Occipito-parietal-frontal * T—A	–14.26	9.61	–33.10	4.58	404.49	–1.48	0.14
Phase 1: Comparison of IAF tACS conditions and sham stimulation conditions on alpha power increase								
(Intercept)	(Intercept)	25.25	5.38	14.71	35.79	19.14	4.69	< 0.001
electrode_pool1	Central-Anterior	–2.71	3.80	–10.15	4.73	628.99	–0.73	0.48
electrode_pool2	Posterior-Anterior	2.42	3.41	–4.25	9.08	628.99	0.71	0.48
condition1	F–A	–3.38	3.54	–10.31	3.56	628.99	–0.95	0.34
condition2	S–A	–8.86	3.54	–15.79	–1.93	628.99	–2.51	0.01
electrode_pool1* condition1	Central-Anterior * F–A	–1.97	9.30	–20.19	16.25	628.99	–0.212	0.83
electrode_pool2* condition1	Posterior-Anterior * F–A	–0.65	8.34	–16.99	15.70	629.01	–0.08	0.94
electrode_pool1* condition2	Central-Anterior * S–A	–0.07	9.30	–18.29	18.15	628.99	–0.01	0.99
electrode_pool2* condition2	Posterior-Anterior * S–A	–13.30	8.32	–29.61	3.018	628.99	–1.60	0.11
tACS modulation specificity on frequency bands for posterior IAF tACS stimulation								
(Intercept)	(Intercept)	18.77	5.65	7.69	29.85	19.17	3.32	0.004
freq_band1	beta—alpha	–21.17	3.69	–34.39	–19.94	454.01	–7.37	< 0.001
freq_band2	theta—alpha	–9.97	3.69	–17.20	–2.74	454.01	–2.71	0.01
electrode_pool1	Occipito-parietal-frontal	–3.71	3.01	–9.61	2.19	454.01	–1.23	0.22
freq_band1 * electrode_pool1	Beta-alpha * occipito-parietal-frontal	–17.85	7.37	–32.30	–3.40	454.01	–2.42	0.02
freq_band2 * electrode_pool1	Theta-alpha * occipito-parietal-frontal	–14.29	7.37	–28.74	0.16	454.01	–1.94	0.05

The table reports mean effects, standard error (SE), 95% confidence intervals (95% CIs), degrees of freedom (df), *t* values and *P*-values.

A = posterior IAF tACS; F = anterior IAF tACS; LMM = linear mixed-effect model;

S = Sham stimulation condition; T = posterior ITF tACS.

### Site of stimulation

#### Comparison across IAF tACS montages and sham on alpha power increase

A LMM was conducted to compare the effects of anterior IAF tACS stimulation condition versus those elicited by the posterior IAF tACS stimulation condition as well as sham stimulation conditionde sites. Refer to Table 2 illustrating this model. We included change ratio (%) as the dependent variable and added fixed effects of condition and electrode pool, as well as the interaction between condition and electrode pool. We included subject id as a random effect. No significant interaction between the condition (anterior tACS versus posterior tACS versus sham) and electrode pools (frontal, central and occipito-parietal) was found, *F*(4, 629) = 1.28, *P *=* *0.28, although main effects of condition reached statistical significance, *F*(2, 629) = 3.20, *P *=* *0.04. Further contrast analyses showed that only the posterior IAF tACS condition (*M* = 33.22, SD = 43.01) elicited a significantly greater percent change from baseline alpha power compared to sham (*M* = 19.84, SD = 36.82), *P* = 0.04, whereas similar comparison between the posterior IAF tACS condition and the anterior IAF tACS did not reveal to be significant (*M* = 26.70, SD = 47.16), *P* = 0.37. Finally, no statistical difference was found between anterior IAF tACS and posterior IAF tACS, *P* = 1.00. See Fig. 3B for overview of results.

### Intensity of stimulation

#### Effects of high versus low intensity alpha tACS stimulation on brain alpha activity

A LMM was conducted to compare the effects of high versus low stimulation intensity on a subsample of participants for the montage PO7PO8 with IAF stimulation condition on α-band power modulation. Refer to Table 3 illustrating this model. We included change ratio (%) as the dependent variable and added fixed effects of intensity [phase 1 (high) versus phase 2 (low)] and electrode pool, as well as the interaction between ratios intensity (phase) and electrode pool. We included subject id as a random effect. No interaction was revealed (*P *=* *0.31) between the two factors intensity (high versus low) and electrode pool (frontal, central and occipito-parietal). No main effect of electrode pool (*P *=* *0.96) comparison nor intensity comparison (*P *=* *0.62) were revealed on alpha power modulation for this analysis.

**Table 3:** LMM analyses results: fixed effects parameters estimates

Names	Effect	Estimate	SE	Lower 95% CI	Upper 95% CI	*df*	*T*	*P*
Phase 1: Alpha power aftereffects: change ratios over time comparison for IAF tACS montages								
(Intercept)	(Intercept)	21.36	5.70	10.19	32.53	19.02	3.75	0.001
condition1	F—A	0.76	2.87	–4.87	6.39	910.06	0.26	0.79
ratios1	var_pre_post60- var_pre_post120	5.34	3.53	–1.57	12.54	910.19	1.52	0.13
ratios2	var_pre_post—var_pre_post120	15.26	3.52	8.36	22.16	910.15	4.33	< 0.001
electrode_pool1	Posterior—Anterior	4.61	2.87	–1.01	10.24	909.92	1.61	0.11
ratios1 * condition1	F—A * var_pre_post60- var_pre_post120	15.48	7.05	1.65	29.30	910.14	2.19	0.03
ratios2 * condition1	F -A * var_pre_post—var_pre_post120	2.46	7.04	–11.34	16.26	909.92	–0.35	0.73
condition1* electrode_pool1	F—A * Posterior—Anterior	–0.50	5.74	–11.74	10.75	909.95	–0.09	0.93
ratios1 * electrode_pool1	var_pre_post60- var_pre_post120 * Posterior—Anterior	–3.03	7.05	–16.84	10.78	909.91	0.43	0.67
ratios2* electrode_pool1	var_pre_post—var_pre_post120 * Posterior—Anterior	1.62	7.04	–12.17	15.41	909.91	–0.23	0.82
ratios1 * condition1* electrode_pool1	F-A * var_pre_post60- var_pre_post120 * Posterior—Anterior	–3.44	14.09	–31.06	24.18	909.95	–0.24	0.81
ratios2 * condition1* electrode_pool1	F-A * var_pre_post—var_pre_post120 * Posterior—Anterior	–1.74	14.07	–29.31	25.84	909.91	–0.12	0.90
Phase 1: Effects of high versus low intensity alpha on alpha power aftereffects, between directly after stimulation, at 60-min and 120-min time points following posterior IAF tACS.								
(Intercept)	(Intercept)	45.02	8.56	28.23	61.80	10.01	5.26	< 0.001
intensity_phase1	Low-High	41.61	2.91	35.91	47.32	672.53	14.30	< 0.001
electrode_pool1	Central-Anterior	10.23	3.79	2.80	17.67	671.99	2.70	0.01
electrode_pool2	Posterior-Anterior	6.42	3.42	–0.28	13.12	672.00	1.88	0.06
ratios1	pre_post60-pre_post	19.26	3.54	12.32	26.20	672.21	5.44	< 0.001
ratios2	pre_post120-pre_post	22.45	3.53	15.53	29.38	672.19	6.36	< 0.001
intensity_phase1 * electrode_pool1	Low-High * Central-Anterior	15.04	7.59	0.17	29.92	671.99	1.98	0.05
intensity_phase1 * electrode_pool2	Low-High *Posterior-Anterior	–8.27	6.84	–21.68	5.13	672.00	–1.21	0.23
intensity_phase1 * ratios1	Low-High* pre_post60-pre_post	62.96	7.08	49.08	76.85	672.21	–1.21	< 0.001
intensity_phase1 * ratios2	Low-High* pre_post120-pre_post	68.77	7.06	54.93	82.62	672.19	9.74	< 0.001
ratios1 * electrode_pool1	Central-Anterior * pre_post60-pre_post	8.65	9.26	–9.51	26.80	671.98	0.93	0.35
ratios1 * electrode_pool2	Posterior-Anterior * pre_post60-pre_post	–13.62	8.35	–29.99	2.75	672.01	–1.631	0.10
ratios2 * electrode_pool1	Central-Anterior * pre_post120-pre_post	11.02	9.26	–7.13	29.17	671.98	1.19	0.24
ratios2 * electrode_pool2	Posterior-Anterior * pre_post120-pre_post	–2.71	8.31	–19.00	13.58	671.98	–0.326	0.74
intensity_phase1 * ratios1 * electrode_pool1	Low—High * Central-Anterior * pre_post60-pre_post	4.61	18.52	–31.70	40.92	671.98	0.25	0.80
intensity_phase1 * ratios1 * electrode_pool2	Low—High * Posterior-Anterior * pre_post60-pre_post	–2.78	16.70	–35.51	29.96	672.01	–0.17	0.87
intensity_phase1 * ratios2 * electrode_pool1	Low—High * Central-Anterior * pre_post120-pre_post	12.64	18.52	–23.66	48.95	671.98	0.682	0.50
intensity_phase1 * ratios2 * electrode_pool2	Low—High * Posterior-Anterior * pre_post120-pre_post	14.34	16.62	–18.24	46.91	671.98	0.863	0.39
Intensity comparison on electrode pools								
(Intercept)	(Intercept)	17.85	4.59	8.86	26.85	10.08	3.89	0.003
phase_nb	2 -1	–1.25	2.53	–6.21	3.71	704.00	–0.49	0.62
electrode_pool1	Central—Anterior	–0.94	3.33	–7.47	5.59	704.01	–0.28	0.78
electrode_pool2	Posterior- Anterior	–0.31	2.99	–6.17	5.54	704.00	–0.11	0.92
phase_1* electrode_pool1	2 -1 * Central—Anterior	8.73	6.67	–4.33	21.79	704.02	1.31	0.19
phase_1 * electrode_pool2	2 -1 * Posterior—Anterior	0.53	5.98	–11.19	12.24	704.05	0.09	0.93

The table reports mean effects, standard error (SE), 95% confidence intervals (95% CIs), degrees of freedom (df), *t* values and *P*-values.

1 = high intensity phase (4–6 mA); 1 = low intensity phase (1 mA); A = Posterior IAF tACS; F = anterior IAF tACS; LMM = linear mixed-effect model; S = Sham stimulation condition; T = posterior ITF tACS.

### tACS modulation specificity on frequency bands

#### Posterior α-tACS stimulation

**Figure 4:**
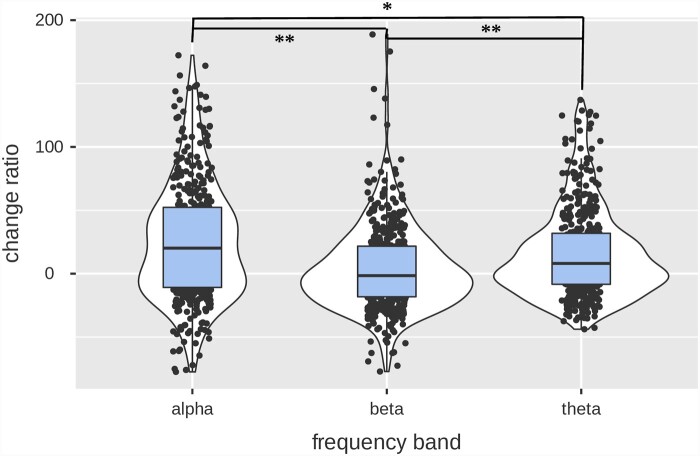
Posterior IAF tACS change ratios of spectral power induced according to frequency bands.

In addition, we conducted another repeated measures LMM to investigate whether posterior alpha tACS stimulation differently impacted occipito-parietal and frontal brain regions across frequency bands of interest. Refer to Table 2 illustrating this model. We included change ratio (%) as the dependent variable and added fixed effects of frequency bands and electrode pool, as well as the interaction between frequency bands and electrode pool. We included subject id as a random effect. We found a statistically significant electrode pool* frequency bands interaction on spectral power change ratios *F*(2, 454) = 3.28, *P *=* *0.04. Contrast analyses showed a significantly greater change ratio of occipito-parietal α-band power (*M* = 34.57%, SD = 51.15) relative to both occipito-parietal β-band power (*M* = -1.44%, SD = 26.41) (*P *<* *0.001) and θ-band power change ratio (*M* = 17.54%, SD = 39.90), *P *=* *0.003. Occipito-parietal θ-band power change ratio was also significantly greater than occipito-parietal β-band power change ratio, with a mean difference of 18.976 (*P *<* *0.001). No statistical difference was found in the change ratio between occipito-parietal α-band power (*M* = 34.57%, SD = 51.15) and frontal α-band power (*M* = 27.64%, SD = 33.24), *P* = 0.90. However, frontal α-band power change ratio (*M* = 27.65%, SD = 33.24) was significantly greater than β-band power change ratio (*M* = 9.41%, SD = 51.03), *P *=* *0.03. Frontal θ-band change ratio (*M* = 24.82%, SD = 29.08) and β-band power change ratio did not differ significantly, *P *=* *0.126. Comparisons between frontal and occipito-parietal electrode pool within each frequency bands revealed no significant difference [theta (mean difference of 7.28%, *P *=* *1.00); alpha (mean difference of -7.00%, *P *=* *1.00) and beta (mean difference of 10.85%, *P *=* *0.57)]. Finally, a main effect of frequency bands was revealed *F*(2,454) = 27.80, *P *<* *0.001. Post hoc comparisons showed a statistically significant higher increase of α-band power (*M* = 31.96%, SD = 45.24) than β-band power (*M* = 2.63%, SD = 37.79), *P *<* *0.001 and θ-band power (*M* = 20.27%, SD= 36.30), *P *=* *0.02. Additionally, change ratios of θ-band power showed much greater increase than β-band power (mean difference = 17.19%), *P *<* *0.001 (Fig. 4B).

### Alpha power aftereffects

#### Power change ratios over time

A LMM was conducted to compare the effects of anterior IAF tACS stimulation versus posterior IAF tACS stimulation on α-band power over time (post-60 min and post-120 min). Refer to Table 3 illustrating this model. We included change ratio (%) as the dependent variable and added fixed effects of ratios (post-60 min and post-120 min), condition and electrode pool, as well as the interaction between ratios, condition and electrode pool. We included subject id as a random effect. No interaction between factors reached statistical significance. No main effect on factors electrode pool (*P *=* *0.11) and conditions (*P *=* *0.79) were observed. However, a main effect of ratios was identified, *F* (2, 910), *P *<* *0.001. Post hoc tests revealed differences in power change ratios when comparing immediate post-tACS measurements with 60-min and 120-min post-tACS measurements on α-band power, independently of stimulation montage. Post-tACS measurements of α-band power revealed a significant decrease when measured post-60-min (mean difference = 9.91%), *P *=* *0.01 and even greater decrease when compared to post-120-min ratios (mean difference = 15.26%), *P *<* *0.001. No significant difference was measured between post-60-min and post-120-min ratios.

#### Correlation analysis of IAF tACS conditions between intensity and percentage of change in alpha power over time

A Spearman’s correlation was computed to assess the relationship between intensity of IAF tACS (anterior and posterior montages) and percentage of change in alpha power at 60-min and 120-min time points. There was a significant, negative correlation between intensity (mA) and percentage of change in alpha power at the 60-min time point, *r*_s_ = –0.23, *P *<* *0.01. However, no correlation between the intensity and the percentage of change in alpha power was found at the 120-min time point *r*_s_ = –0.13, *P *=* *0.17.

### Effects of high versus low intensity alpha on alpha power aftereffects

**Figure 5:**
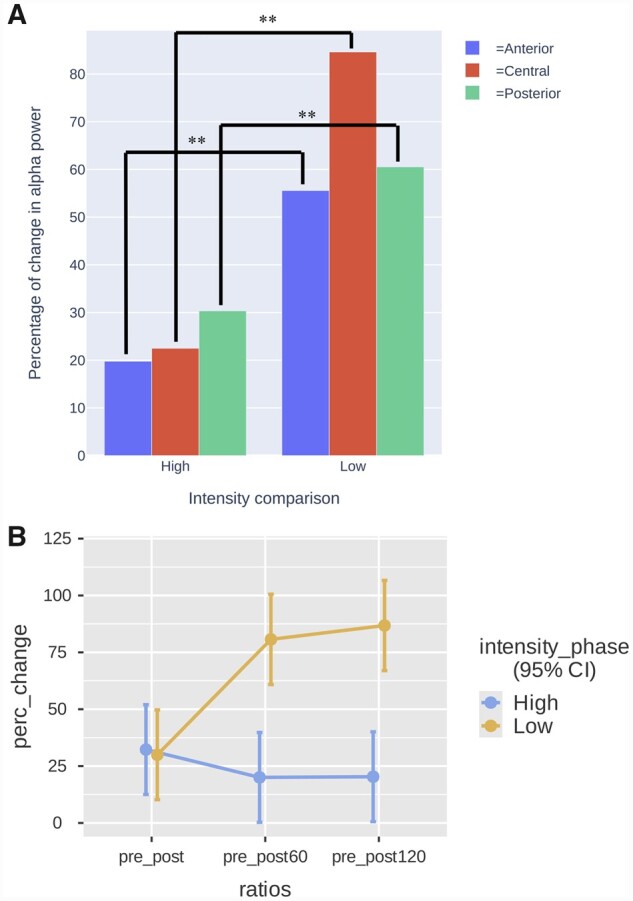
Comparison of change in alpha power between intensity and time. Comparison of alpha power increase ratios between high and low posterior IAF tACS conditions between the three cortical sites (independently of time) in **A.** Comparison of alpha power increase ratios between high and low posterior IAF tACS conditions at three time points (directly after stimulation, at 60 min and at 120 min) in **B.**

Another LMM was conducted to compare the effects of IAF tACS of different intensities (high versus low) on α-band power modulation over time at the 60-min and 120-min time points. Refer to Table 3 illustrating this model. We included change ratio (%) as the dependent variable and added fixed effects of intensity [phase 1 (high) versus phase 2 (low)], ratios (time points) and electrode pool, as well as the interaction between ratios, intensity (phase) and electrode pool. We included subject id as a random effect. We found a statistically significant intensity_phase * electrode pool interaction on spectral power change ratios *F*(2, 672) = 5.86, *P *=* *0.003 and a statistically significant intensity_phase * ratios interaction on spectral power change ratios *F*(2, 672) = 58.59 *P *<* *0.001. For the intensity_phase*electrode pool interaction (Fig. 5A), contrast analyses showed a significantly greater increase in alpha power for the low intensity condition relative to the high intensity condition for all three cortical sites [occipito-parietal low (*M* = 54.37%, SD = 48.33) versus occipito-parietal high (*M* = 30.35%, SD = 52.79), *P *<* *0.001; central low (*M* = 74.37%, SD = 60.99) versus central high (*M* = 22.50%, SD = 37.08), *P *<* *0.001 and frontal low (*M* = 40.51%, SD = 49.43) versus frontal high (*M* = 19.79%, SD = 36.87), *P *<* *0.001]. For the intensity_phase*ratios interaction (Fig. 5B), contrast analyses showed a significantly greater increase in alpha power for the low intensity condition relative to the high intensity condition over time when compared at 60-min [low pre_post60 (*M* = 75.17%, SD = 56.91) versus (*M* = 20.40%, SD = 38.70), *P *<* *0.001] and 120 min [low pre_post120 (*M* = 82.87%, SD = 50.68) versus (*M* = 21.08%, SD = 48.95), *P *<* *0.001] time points. Furthermore, the increase of alpha power is significantly greater over time when compared to the initial ratio (pre_post) for the low intensity condition [low pre_post (*M* = 30.16%, SD = 33.32) versus low pre_post60 (*M* = 75.17%, SD = 56.91), *P *<* *0.001; low pre_post120 (*M* = 82.87%, SD = 50.68), *P *<* *0.001]. This increase of alpha power was not observed for the high intensity condition [high pre_post (*M* = 34.49%, SD = 45.69) versus high pre_post60 (*M* = 20.40%, SD = 38.70), *P *=* *0.200; high pre_post120 (*M* = 21.08%, SD = 48.95), *P *=* *0.24].

### Comfort assessment

#### Sensation and side effects assessment phase 1

Follow-ups on potential side effects resulted in two participants reporting a headache at the end of day 1 (after having spent 7 h at the laboratory) and 6 participants reporting fatigue upon completing the study protocol. Phosphene thresholds were documented but not used as a criterion to set stimulation intensity. Of the 3 active conditions, anterior IAF tACS elicited phosphenes in 19 participants (95% of overall sample). In contrast, the posterior tACS montage elicited phosphenes in 8 participants (40% of the overall sample) when administered the IAF condition relative to 5 participants (25% of overall sample) for the ITF tACS condition.

#### Sensation and side effects assessment phase 2 (low intensity stimulation only)

No side effects were reported at follow-ups. Phosphene thresholds were documented but not used as a criterion to set stimulation intensity. Low-intensity IAF tACS (PO7PO8) elicited phosphenes in 4 participants (20% of overall sample).

## Discussion

This study sought to characterize the contribution of important tACS stimulation parameters in modulating endogenous alpha power. More specifically, the impact of stimulation intensity, site and frequency that are highly heterogeneous in practice, was investigated via three active tACS conditions and one sham stimulation condition. This initiative stems from the recognized needs to standardize practice in neurostimulation research so as to improve replicability and optimize intervention response. While previous tACS studies contrasted current density, stimulation duration, electrode size and montage parameters, the current study innovates by testing the impact of higher stimulation intensities using wide circular tACS electrodes on the modulation of alpha band activity. This constitutes an important step towards improving tACS practices for alpha power modulation.

In this novel assessment of suprathreshold tACS stimulation intensity, we found excellent tolerability in addition to demonstrating equivalent within-subject alpha power modulation across tACS intensity conditions. However, contrary to our expectations, low intensity IAF tACS induced significantly greater aftereffects on alpha power when tested at both 60- and 120-min post-stimulation.

Moreover, this within-subject study design investigated the capacity of IAF tACS stimulation near alpha generators (occipito-parietal) to modulate alpha power relative to anterior stimulation, the latter being selected for the preponderance of alpha power relative to other frequency bands. We found that only the posterior IAF tACS montage elicited significant increases in alpha power over both frontal and occipito-parietal cortical sites relative to the sham stimulation condition. In sharp contrast, the anterior IAF tACS montage did not significantly modulate alpha power relative to the sham stimulation condition. This finding suggests that induced effects of IAF tACS montage location on alpha power is influenced by the relative proximity to alpha generators. Given that alpha activity originates and dominates over occipito-parietal brain areas (Schürmann and Başar, 2001, Romei *et al.*, 2008a; Başar, 2012; Helfrich *et al.*, 2014), the latter findings extend current knowledge on alpha tACS effects as it shows that significant tACS stimulation effects on endogenous alpha power can be observed at a distant brain region from tACS stimulation location. In NIBS research, the cortical site of stimulation is a central parameter (Dmochowski *et al.*, 2011, 2017) often selected by investigators in order to modulate underlying neuronal networks and function (Feurra *et al.*, 2011, Neuling *et al.*, 2013a; Mehta *et al.*, 2015; Vosskuhl *et al.*, 2016). Interestingly, neighbouring frequency bands each contributing to a given neural network can behave in two different ways. On the one hand, frequency bands can be associated with a divergent brain state and therefore compete with one another. However, frequency bands may coexist within shared or different networks leading to mutual synergy and influence (Klimesch, 1999; Engel *et al.*, 2001; Csicsvari *et al.*, 2003; Buzsáki and Draguhn, 2004; Helfrich *et al.*, 2016). Mutual and/or competing interaction among frequency bands from a neural network can help explain the intricate effects of tACS stimulation at a given frequency on brain oscillations of varying frequencies. To add to the complexity of neuronal connectivity, oscillations at a given frequency are recorded from various neuronal networks throughout the brain and contribute to distinct brain functions. In particular, posterior alpha, in awake at-rest individuals, is associated with brain functions—namely visual system functions, visual attention and perception, gating and information processing (Klimesch, 1999, 2012; Palva and Palva, 2007; Bazanova and Vernon, 2014; Hari and Puce, 2017) that are distinct from those associated with anterior alpha activity—those being associated with top-down control regulation in perception and attention (Schürmann and Başar, 2001, Romei *et al.*, 2008a; Thut *et al.*, 2012; Lange *et al.*, 2013; Misselhorn *et al.*, 2019). These results support the known extensive, interrelated connectivity between posterior and anterior neural networks, especially those originating from the visual system, which is by far the dominant human sense (Thomas Yeo *et al.*, 2011; Parks and Madden, 2013; Vossel *et al.*, 2014). In sharp contrast to anterior tACS stimulation, the latter finding suggests that positioning the IAF tACS montage over posterior brain regions could reveal to be efficient in modifying alpha activity involved in occipito-parietal brain functions such as visual-spatial attention (Zaehle *et al.*, 2010, Neuling *et al.*, 2013a; Strüber *et al.*, 2015; Vossen *et al.*, 2015; Dowsett and Herrmann, 2016; Kasten *et al.*, 2016; Stecher *et al.*, 2017; Fuscà *et al.*, 2018; Stecher and Herrmann, 2018; Schwab *et al.*, 2019). Moreover, this significant modulation of alpha power induced by the posterior IAF tACS montage also suggests a modulation of spectral power occurring distally to the stimulation site. This finding could reveal to be clinically important following brain damage as recovery of related brain function could nonetheless be facilitated using a tACS montage strategically positioned away from damaged areas.

Moreover, results from the current study reaffirm the specificity of alpha tACS stimulation effects to brain oscillations within the corresponding spectral band. For the posterior IAF tACS condition, while an overall dominance of alpha power increase was observed, the alpha-specific effect was only recorded from electrodes near the stimulation site. Indeed, posterior IAF tACS induced alpha power increases significantly greater than beta and theta power increases at both the occipito-parietal site and the frontal cortical site. This finding provides additional knowledge on the understudied impact of IAF tACS on other frequency bands. This finding is also consistent with results from Helfrich *et al*. (2014), showing a specific increase of alpha power when compared to theta and beta spectral power following a 20-min and 10 Hz tACS session applied over the occipito-parietal cortex (Helfrich *et al.*, 2014).

Another important finding from this study is that under fixed tACS montage position (near alpha generators) and stimulation intensity, modulation of endogenous alpha power is significantly greater when stimulating at alpha frequency relative to theta frequency. This finding is important as it demonstrates, at least for alpha power, that preferential effects of tACS within a targeted frequency band is most likely to be achieved when tACS montage is positioned over cortical regions where the targeted endogenous frequency dominates.

Secondary analyses also showed that stimulation sites influence long term aftereffects of alpha tACS stimulation. Contrary to previous results from Kasten *et al*. (2016), posterior IAF tACS stimulation in this study did not induce aftereffects on alpha power when measured either at 60-min or 120 min post-tACS. Moreover, when posterior IAF tACS was compared with the anterior IAF tACS, no difference between conditions was observed on the decrease of alpha power neither at the 60-min nor the 120-min time points. Given that IAF tACS had been applied at a much higher intensity in most participants, this result pattern suggests that higher tACS intensity stimulation does not improve the durability of IAF tACS effects. On the contrary, correlational analyses rather suggested that stimulation intensity under IAF tACS condition negatively correlated with alpha power modulation when measured at the 60-min time point. The latter finding could reveal to be important as it suggests that individually adjusted stimulation intensity, whether based on phosphene thresholds, perception or comfort level, seems to introduce unwanted tACS response variability, particularly when assessing its durability. Results from the second study phase conducted in participants who well tolerated high-intensity posterior IAF tACS (4–6 mA) further consolidated this observation. Indeed, when retested a few weeks later with a fixed, low-intensity posterior IAF tACS (1 mA), participants showed no greater immediate benefit of high IAF tACS stimulation intensity versus low intensity on alpha power modulation. However, when we contrasted IAF tACS’ aftereffects at both 60-min and 120-min time points, the initial increase of alpha power recorded immediately after IAF tACS stimulation was found to have increased significantly further when measured at the 60-min time-point post-tACS only for the low intensity condition, independently of brain regions. Importantly, this sizable increase in alpha power for the 1 mA IAF tACS condition not only persisted over time, but it was found to be considerably greater at the 120-time-point. These findings show that relative to IAF tACS applied at never-before-seen high intensities (> 4–6 mA), lower (1 mA) stimulation intensity was more effective in inducing durable increases in alpha power beyond time points previously investigated (Kasten *et al.*, 2016). In sum, these findings highlight the efficacy of a fixed 1 mA IAF tACS, as opposed to stimulating at higher intensities, to modulate endogenous brain oscillations of the stimulating frequency band. This shows that lower (1 mA) intensity of stimulation led to a much superior rise in alpha power over time than higher intensities (> 4–6 mA), beyond time points previously investigated (Kasten *et al.*, 2016).

This result pattern was found consistent for alpha activity recorded from both frontal and occipito-parietal cortical sites. This second phase of our study expands current knowledge on alpha tACS effects as it shows that: (i) significant IAF tACS stimulation effects can be observed on alpha power at both high and low intensities when recorded within minutes of tACS stimulation, but (ii) low intensity shows much stronger and longer aftereffects than high-intensity IAF tACS, at least, when the tACS montage is set on the occipito-parietal site. Synaptic plasticity mechanisms specifically linked to spike-timing-dependent plasticity have been shown to be involved in lasting aftereffects on alpha power (15–17.86). This result pattern is consistent with Batsikadze *et al*. (2013) study in which increasing transcranial direct current stimulation intensity over the primary cortex reversed expected cathodal stimulation effects (i.e. increased excitability rather than its usual cortical inhibition) (Batsikadze *et al.*, 2013). Nonetheless, tACS implicates different mechanisms than transcranial direct current stimulation, mainly by the omission of directional voltage and the result of continuous reversing of electron flow at the cellular membrane (Tavakoli and Yun, 2017). Although using distinct frequency and site parameters, other tACS studies have previously reported intensity-dependent effects. For example, in a study conducted by Moliadze *et al*. (2012), tACS applied over M1 at 140 Hz showed intensity-dependent aftereffects. In the latter study, intensity set at 0.2 mA did not induce any effect, 0.4 mA led to inhibition, whereas 0.6 and 0.8 mA did not induce significant effect (Moliadze *et al.*, 2012). Antal and Paulus (2013) suggested that tACS applied at 0.4 mA could inhibit intracortical facilitatory effects on corticospinal motoneurons (Antal and Paulus, 2013). Interestingly, Dan and Poo (2006) conducted a stimulation study on rats showing that low alternating current stimulation intensity only modulated spike timing. At higher intensities, alternating current stimulation was found to also modulate spiking rates (83). Given that tissue resistivity in animal models are analogous to those of humans (84), the demonstration that low-intensity transcranial electrical stimulation is subjected to extensive dispersion through tissue and skull has cast some doubt on the effectiveness of tACS effects in modulating cortical activity (26). Accordingly, recent papers by Tavakoli and Yun (2017) and Lee *et al*. (2017), presented current density modelling data associated with TES. However, findings from this study show a significantly greater increase in alpha power over time for the 1 mA IAF tACS condition relative to the high-intensity IAF tACS condition. Effects on later time points thus appear much more significant than immediately after stimulation. This finding must be taken into consideration for future neurostimulation protocol design.

## Limitations

One downside of stimulating at higher stimulation intensities, particularly when conducting a within-subject study design, is that the subject’s blindness to the sham condition was not possible. Although we fully acknowledge that this may have potentially introduced a certain bias, one can question whether a conventional sham condition, obtained with the progressive interruption of current flows, can be achieved with any TES techniques. As reported in many previous studies, dermic sensitivity thresholds to TES are attained even at very low intensities (< 1 mA) (Romei *et al.*, 2008b; Zaehle *et al.*, 2010; Neuling *et al.*, 2012; Foerster *et al.*, 2013; Helfrich *et al.*, 2014; Vossen *et al.*, 2015; Kasten *et al.*, 2016) such that participants will most often notice current interruption. Moreover, action mechanisms underlying tACS modulation effects on brain oscillations are still subject of discussion, in part due to recent studies most partly attributing induced effects to extraneous sensory stimulation (Asamoah *et al*., 2019). The transcutaneous stimulation theory premise stems from the demonstration that electric fields from the skin can be much stronger than those recorded at the cortical level such that recorded electric activity modulation with scalp electrodes would rather be the result of the rhythmic excitation of peripheral nerves mediating the effect (Asamoah *et al*., 2019). Our study used various parametric comparisons that limit the weight of this potential confounding factor. Contrary to the expected linear increase of alpha effect with increasing stimulation intensity at the transcutaneous level, our tACS findings rather indicate persistently lower alpha activity modulation from high-intensity stimulation. Also, another confounding factor is the potential mediating role of optic nerve stimulation on tACS modulation effects. Indeed, electrical stimulation of the optic nerve was shown to result in neural entrainment (Raco *et al*., 2014). As mentioned in the results section, the anterior tACS montage induced phosphene among 95% of the participants by opposition to 40% of the participants for the posterior IAF tACS condition. However, stimulation via the anterior IAF tACS montage did not show a significant increase in alpha power whether compared to sham or posterior IAF tACS montages. As only the posterior IAF tACS montage induced significant alpha power modulation relative to sham stimulation, the contribution of optic nerve stimulation in induced posterior tACS stimulation effects is likely to be limited.

Another limitation to our study had to go with study materials, specifically the tACS electrodes used. Our electrode dimensions were unconventional and covered larger surfaces, as opposed to focal surfaces, which is known to reduce spatial locality of stimulated brain regions (Dmochowski *et al.*, 2011). Another commonly reported issue with tACS neuromodulation paradigms is that the induction of phosphenes could potentially amplify alpha activity (Romei *et al.*, 2008a; Schwiedrzik, 2009; Kanai *et al.*, 2010; Herrmann *et al.*, 2013). Furthermore, a recent paper by Asamoah *et al*. (2019) suggested that tACS administration induces transcutaneous stimulation via peripheral nerves as opposed to stimulating cortical neurons. In the latter paper, the rhythmic modulation of peripheral nerves was suggested to drive rhythmically cortical neurons. Although this represents an intriguing alternative explanation for tACS action mechanisms, further validation efforts would be needed (Asamoah *et al.*, 2019). Moreover, phosphenes are more common when the tACS montage is positioned over fronto-central areas relative to occipito-parietal areas (Schutter and Hortensius, 2010). However, the retinal or cortical origins of phosphenes were unlikely to have influenced current study findings as EEG data were acquired offline, therefore considerably diminishing the potential influence of transient phosphenes on measured activity. Finally, our study design included two blocks of tACS stimulation within the same day, when a more conservative approach would have been to wait for a few days between tACS stimulation conditions. However, given results from several studies reporting tACS aftereffects lasting up to a maximum of 70 min (Neuling *et al.*, 2013a; Strüber *et al.*, 2015; Kasten *et al.*, 2016), our study design included three control steps: (i) we made sure to include a rest period of at least 3 h between tACS sessions; (ii) we monitored tACS aftereffects of alpha power levels with 5-min resting EEG recordings at 60-min and 120-min post-stimulation and (iii) Block randomization was used in order to counterbalance tACS conditions across participants. Finally, it is to note that psychomotor vigilance task performance during tACS stimulation is likely to have facilitated alpha modulation via a state-dependent effect. This notion is in keeping with previous demonstrations that attention and preparedness for external input required to perform the vigilance task are known to increase alpha-band activity as a result of increasing information processing demand during tACS stimulation (Jann *et al.*, 2010; Mierau *et al.*, 2017). However, the inclusion of a sham stimulation condition allowed to isolate the modulating effect of the psychomotor vigilance task on alpha activity, whereas the ITF (theta) condition provided a systematic comparison by frequency of stimulation.

## Conclusion

Our study provides key reference parameters to improve modulation of alpha power using tACS. First, the 2- to 3-fold increase in tACS stimulation intensity was well tolerated and validated the use of stimulation intensity above phosphene and sensation thresholds or > 2 mA in alpha tACS stimulation protocols. Stimulation frequency set according to IAF was also shown to efficiently modulate alpha activity only when tACS montage was positioned near alpha generators. Additionally, this posterior IAF tACS montage induced an overall increase in alpha power diffusely across frontal, occipito-parietal and central regions of the cerebral cortex. Finally, 1 mA tACS showed much higher efficiency than high intensity IAF tACS in inducing long-term aftereffects on alpha power modulation (up to 2 h), as it was associated to a 3-fold increase in alpha power relative to alpha power measured immediately after IAF tACS stimulation. Effects of a fixed 1 mA IAF tACS on alpha power measured at 60-min and 120-min time points are considerably greater than that recorded immediately after stimulation, suggesting that IAF tACS is effective in triggering long term neuroplastic changes. Modulation of brain oscillations via tACS beyond the initial synaptic plasticity mechanisms holds great therapeutic potential, especially in light of its flexibility of use from different brain sites. This finding must be taken into consideration for future neurostimulation protocol design.

## Abbreviations

FFTs =: fast fourier transforms
IAF =: individual’s alpha frequency
ISF =: individual stimulation frequency
ITF =: individual’s theta frequency
LMM =: linear mixed-effect model
NIBS =: non-invasive brain stimulation
rTMS =: repetitive transcranial magnetic stimulation
tACS =: transcranial alternating current stimulation
tDCS =: transcranial direct current stimulation
VAS =: visual analogue scale
